# Techno-economic and environmental analysis of organic municipal solid waste for energy production

**DOI:** 10.1016/j.heliyon.2024.e31670

**Published:** 2024-05-21

**Authors:** Samina Alam, Md. Rokonuzzaman, Kazi Sajedur Rahman, Akramul Haque, Md Shahariar Chowdhury, Tofan Agung Eka Prasetya

**Affiliations:** a Department of Electrical and Electronic Engineering, Premier University, Chittagong 4203, Bangladesh; b School of Engineering and Advanced Engineering Platform, Monash University Malaysia, Jalan Lagoon Selatan, 47500 Bandar Sunway, Selangor, Malaysia; c Solar Energy Research Institute, Universiti Kebangsaan Malaysia, 43600 Bangi, Selangor, Malaysia; d Health and Environmental Research Center, Faculty of Environmental Management, Prince of Songkla University, Hat Yai 90110, Songkhla, Thailand; e Faculty of Vocational Studies, Universitas Airlangga, Surabaya 60285, East Java, Indonesia

**Keywords:** Sustainable energy, Waste management, Waste-to-energy technologies, Circular economy in waste management, Biogas production

## Abstract

Addressing the critical conundrum of escalating municipal solid waste (MSW) and shrinking landfill spaces in urban areas, this research pioneers a sustainable approach for Bangladesh by exploring the potential of biogas production from MSW. Distinctly, it fills the research gap by providing a detailed techno-economic and environmental analysis of decentralized fixed-dome anaerobic digestion facilities in the urban context of Chittagong, Bangladesh, a domain previously underexplored. Our findings demonstrate the feasibility of converting MSW into a renewable energy source, offering an innovative solution that simultaneously tackles waste management and energy generation challenges. Each proposed plant showcases the capability to generate 536 m³ of biogas daily, sufficient to power a 50 kW gas engine and supply 44 households, thereby contributing significantly to urban waste reduction and CO_2_ emissions mitigation by approximately 500 tons monthly. The economic analysis reveals an attractive investment payback period of two years, underscoring the model's viability and its potential as a replicable framework for similar urban settings grappling with waste management crises. This study not only bridges a critical knowledge gap but also introduces a novel, sustainable waste-to-energy model, marking a pivotal step towards achieving energy security and environmental sustainability in developing nations.

## 1 Introduction

The growing amount of municipal solid waste (MSW) and its detrimental environmental effect compelled the world to focus on the careful management of MSW. According to the World Bank, humans produce an astonishing 2 billion tons of MSW annually [1]. By 2050, 3.4 billion tons of trash are anticipated to be made worldwide [1]. It is necessary to discard the traditional linear economic paradigm, which specifies the idea of ‘take-make-dispose’, to reduce the amount of MSW produced annually. Instead, the circular economic (CE) model will be the sustainable solution, where WtE will play a significant role. WtE is an efficient energy substitute for its economic sustainability and financial feasibility [2]. Researchers in Ref. [3], observed the impacts of WtE over the MSW of Rajshahi City Corporation, where they showed that, over 159.40 MWh can be produced from the daily collection of MSW. Besides this energy production, WtE can remarkably decrease the net volume of waste that must be dumped in landfills. MSW includes biomass material, such as food, wood squander, paper, garments clothes, plastics, elastic and other daily utilized and disposed of materials [4]. In Nigeria, it is expected that, biomass and MSW will be the prospects for mitigating the intensifying energy demand [5]. In case of WtE is not chosen for waste management, it will open the doorway of various problems as over 90 % waste in developing nations is frequently dumped in uncontrolled landfills or burned in the open spaces [6]. MSW accounts for 11 % of all methane emissions worldwide [7]. Besides methane emissions, the decomposition of MSW generates greenhouse gases (GHGs), which are also responsible for climate change. It is approximated that around 3.3 billion tons CO_2_ is emitted yearly in the air due to the global food waste (FW) treatment [8]. Improperly handled, discarded, or burned waste endangers human health, damages the climate, and prevents economic growth in the countries. Therefore, to achieve the sustainable development goals, it is essential to have adequate and effective solid waste management.

Among various waste treatment methodologies, AD is considered more effective than traditional disposal processes, not only in the case of energy conversion but also in economic feasibility [9,10]. Concurrently, it can accomplish efficient waste treatment and produce biogas and electricity [9,11,12]. Implementing AD, a country like China is producing renewable energy [13]. It is found that AD is the most suitable technology for Chittagong, Bangladesh [14]. For the huge prospect of AD technology in Bangladesh, several research studies have been done. Authors in Ref. [15], applied the AD process for the sugarcane-related byproducts and poultry farms droppings overlooking most types of MSW, where AD suits best for the range of 11–15 % of total solid. Some researchers studied biogas generation from cattle feedstock, poultry feedstock and rice straw bedding [16,17]. In contrast, another group of researchers optimized the total solid in kitchen waste to implement an efficient AD process [18]. Researchers in Ref. [19] worked on MSW but provided the probable renewable energy production without any analysis methods. The authors in Ref. [20] mostly focused on biogas production with detailed design and implementation, except for the AD process. The capability of hydrogen gas generation has been studied using the AD process [21]. In Rajshahi City Corporation area, the AD processing of MSW has been investigated [3], but the detailed design of the digester was not explored. This article responds to critical issues created by enormous growth of MSW waste by investigating the feasibility of anaerobic digestion technology to convert MSW into biogas, a renewable energy source. Our research stands at the intersection of waste management and energy production, offering a novel solution that not only mitigates waste disposal problems but also contributes to the renewable energy mix of Bangladesh. What sets this study apart is its focus on the techno-economic and environmental analysis of establishing decentralized anaerobic digestion facilities in a specific urban context—Chittagong, Bangladesh. Unlike previous studies that have broadly addressed waste-to-energy (WtE) technologies, this article delves into the localized feasibility, economic viability, and environmental benefits of biogas production from MSW. The innovation lies in our comprehensive approach, combining detailed site-specific analysis with broader implications for sustainable urban development. By presenting a model for transforming waste into a valuable resource, we contribute to the global discourse on sustainable waste management and renewable energy generation, offering actionable insights for policymakers, urban planners, and researchers alike. In summary:

• The ingredients and the quality of the MSW were examined.
• A digester of the specific size, which can be made locally, has been designed.
• Calculated the amount of biogas generation and equivalent electrical energy generation.
• Accomplished the financial analysis to set up a plant while considering the payback periods.
• Studied the environmental effect by reducing MSW by 90 % and mitigating GHG by 93 % compared to the base case (coal fired power plant).

The unique contribution of this research lies in its application of waste management technologies in the context of a developing nation, offering detailed techno-economic analysis and highlighting the importance of local customization and design innovation. By providing valuable insights into the feasibility, cost-effectiveness, and environmental impacts of AD technology, the study arms policymakers and stakeholders with critical information necessary for assessing its viability for sustainable waste management and energy production. Illustrating economic incentives, including substantial cost savings and the generation of revenue from energy sales, the research underscores AD's pivotal role in driving sustainable economic progress, promoting energy independence, and facilitating job creation, all while alleviating environmental degradation. This research presents a sustainable waste management approach that significantly reduces environmental pollution by converting organic waste into biogas instead of landfills. This method mitigates methane emissions, a potent greenhouse gas, contributing to cleaner air and a healthier environment for communities. The conversion of organic waste into biogas and electricity provides an alternative and renewable energy source, addressing community energy demands. This reduces reliance on non-renewable energy sources and improves energy accessibility, particularly in areas with scarcity. Implementing AD also promotes efficient and sustainable waste management reducing the waste needed for landfilling and encouraging responsible waste disposal and recycling within communities. Proper waste management directly impacts public health by minimizing the risks associated with uncontrolled waste disposal. AD reduces hazardous practices, leading to cleaner surroundings and improved public health conditions. This research also aligns with the principles of a circular economy by converting waste into valuable resources like biogas and electricity, promoting the reuse of organic matter, and reducing the linear ‘take-make-dispose’ model. This approach contributes to a more sustainable and resource-efficient system, fostering a circular economy mindset. The article also discusses the growing challenge of MSW management and proposes a sustainable solution through WtE technologies. It emphasizes the environmental impacts and potential energy generation from source-separated organic waste, aligning with global sustainability goals. The research advances AD technology for MSW treatment, highlighting its energy generation potential and economic feasibility compared to traditional disposal methods. The research offers practical design parameters for AD systems, financial evaluations, and site selection considerations, aiding policymakers and stakeholders in making informed decisions for implementing waste management solutions. The article holds global relevance as it addresses a universal issue of MSW management. It offers a blueprint for integrating technology, economics, and environmental concerns in seeking effective waste-to-energy solutions. Focusing on WtE via AD, this methodology tackles the global challenge of MSW management and significantly contributes to various Sustainable Development Goals (SDGs) [22]. The main focus is achieving Goal 7 by prioritizing WtE through anaerobic digestion. This approach promotes using renewable and readily available energy sources while reducing dependence on fossil fuels. Furthermore, it contributes to the achievement of Goal 11 by effectively overseeing the disposal of municipal solid waste, reducing landfill trash, and promoting cleaner urban surroundings. The transition from a linear to circular economic model, following Goal 12, focuses on responsible consumption through the conversion of trash into energy, emphasizing sustainable waste management practices. The initiative effectively addresses Goal 13 by actively reducing methane emissions and greenhouse gases during waste decomposition, significantly contributing to greater climate mitigation efforts. Moreover, it is in line with Goal 9 as it demonstrates ingenuity in waste management methods, namely in the design and effectiveness of digesters. The research highlights the importance of meticulous financial planning and investment in waste-to-energy plants, recognizing its initial expenses. This article is structured to guide the reader through a comprehensive analysis of the feasibility, economic viability, and environmental benefits of utilizing anaerobic digestion for biogas production from municipal solid waste in Bangladesh. Following the introduction in Section 1, Section 2 outlines the Research Methodology, detailing the study area, data collection processes, and the analytical methods employed. Section 3, Results, presents the key findings related to biogas yield, energy production capacity, and potential environmental impacts. Section 4, Discussion, interprets these results in the context of Bangladesh's waste management and energy challenges, comparing them with global practices and highlighting the study's innovation and significance. The Financial Analysis in Section 5 examines the economic feasibility and investment returns of the proposed anaerobic digestion facilities. Section 6 delves into the Environmental Impact Assessment, evaluating the project's potential to reduce greenhouse gas emissions and contribute to sustainable waste management. Finally, Section 7, Conclusions and Future Directions synthesizes the study's findings, discussing their implications for policy, practice, and further research.

## 2 Research methodology

**Fig. 1:**
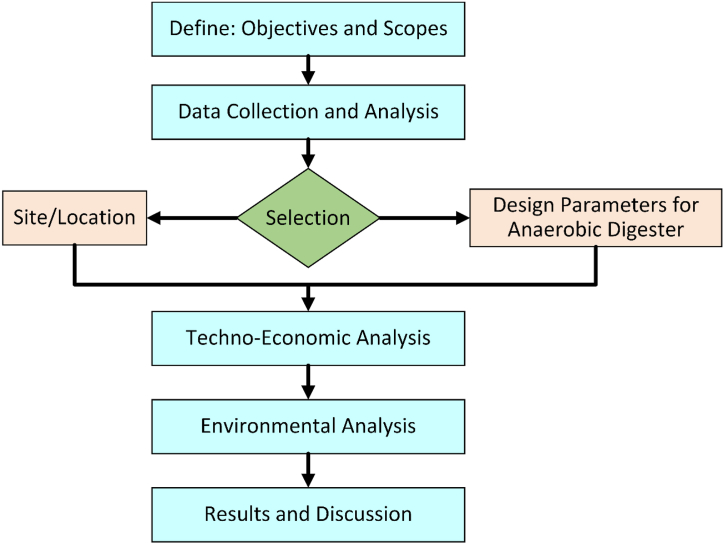
Flowchart of research methodology.

The article explores the design, sizing, and feasibility of an anaerobic digester system for converting organic municipal solid waste into energy. It uses RETScreen software for techno-economic analysis and conducts an environmental impact assessment. The process optimization is discussed, focusing on feedstock composition, dilution ratios, and biogas production potential. The system's capability to produce biogas for electricity generation and household use is evaluated. The article also discusses the technical feasibility and challenges of implementing anaerobic digestion technology in developing countries, focusing on local expertise, materials availability, construction, and maintenance. The flow chart of the research methodology of this paper is shown in Fig. 1.

The article offers valuable insights into process engineering design and implementation in waste-to-energy projects. The article discusses the growing challenge of MSW management and proposes a sustainable solution through WtE technologies.

### 2.1 Data collection and analysis

**Fig. 2:**
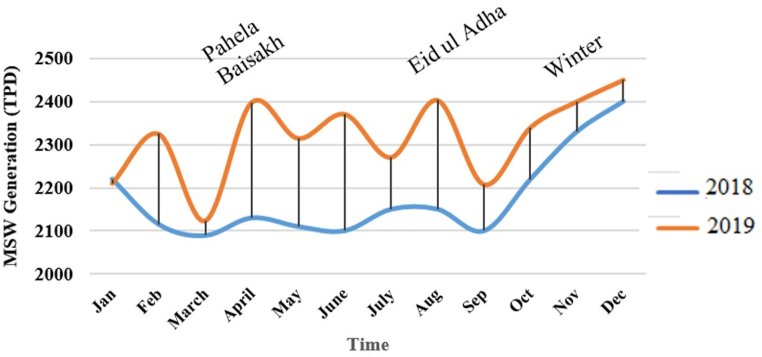
Variation of MSW generation with season.

The quantity and quality of waste in Chittagong, Bangladesh, have been analyzed using primary data from the Chittagong City Corporation authority. The annual waste generation rate ranged from 2000 tons per day (TPD) to 2400 TPD from 2016 to 2019, with an upward trend. The waste generation rate increased by over 500 TPD over the last decade (Fig. 2). However, the coronavirus effect significantly dropped waste generation in 2020, especially during the lockdown period. After the lockdown, waste generation rose again, with a peak in August. The city's waste generation also varies with season and festival, with higher vegetable production in winter increasing waste production by around 5 %. Waste generation also rises during festivals like 'Pahela Baishakh' in April and 'Eid ul Adha'. The waste composition was determined using the quartering process, which was done within four to 5 h to minimize degradation. The waste was then divided into four sections: A, B, C, and D, with the remaining two sections omitted. Repeating these steps yielded almost 15 kg of waste.

**Fig. 3:**
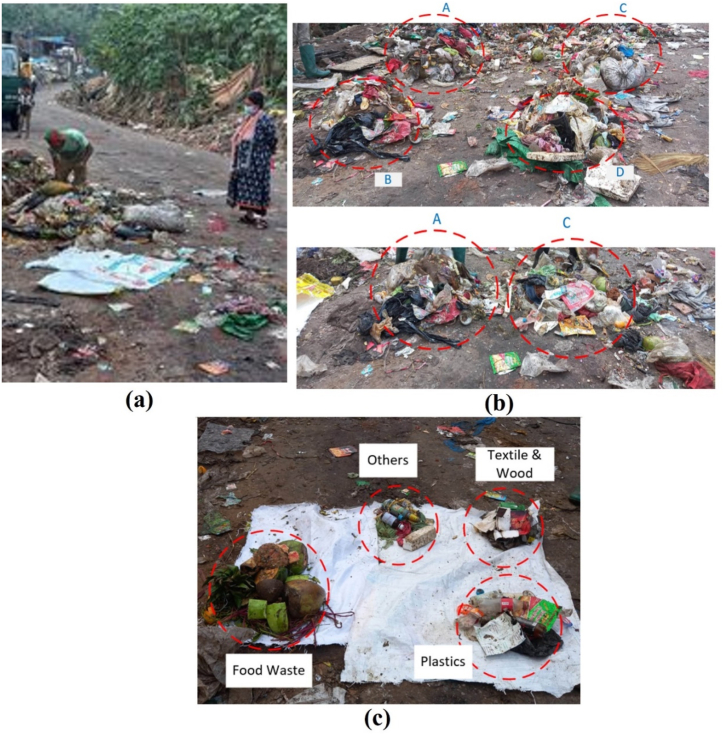
MSW sampling.

The whole waste collection and sorting process has been shown in Fig. 3(a–c). The quantity and quality of waste in Chittagong, Bangladesh, have been analyzed using primary data from the Chittagong City Corporation authority shown in Fig. 3a. Following reduction, the decreased sample was physically divided into various categories, including food waste, fabric, paper, plastic and others. As seen in Table 1, it was discovered that food makes up a sizable amount of the waste (around 80 %). Proximate analysis of the collected sample showed that it included 79.3 % moisture, 17.3 % volatiles, 2.2 % ash, and 0.9 % fixed carbon.

**Table 1:** Municipal solid waste composition of Chittagong city, Bangladesh.

Waste	Ref. [8]	Ref. [24]	Ref. [25]	Ref. [26]	Present Study
Food/Domestic	77.7	73.6	62	72	78
Textile &Wood	5.33	2.1	4	5	5
Paper	4.84	9.9	3	5	4.5
Pack	–	–	9	6	–
Can	–	–	9	4	–
Plastic	7.35	2.8	2	3	7.5
Glass	–	1	5	3	–
Rock	–	–	6	2	–
Leather & Rubber	–	–	–	–	–
Metal	0.44	2.2	–	–	0.5
E-waste	0.64	–	–	–	–
Others	3.74	8.4	5	3	4.5

The study conducted a proximate analysis at the Asian Institute of Technology (AIT) laboratory to understand the composition and characteristics of municipal solid waste used in AD. The analysis involved drying a sample in an oven at 105 °C until a constant weight was achieved, determining the moisture content. Volatile matter was measured by heating the sample in a muffle furnace at 950 °C without air, recording the loss in weight. Ash content was determined by burning the remaining material post-volatile matter analysis in a muffle furnace at 575 °C until a constant weight was achieved. The fixed carbon content was calculated by subtracting the sum of the percentages of moisture, volatile matter, and ash from 100 %. The results of the proximate analysis provided essential data on the waste composition, particularly moisture and organic content, which are critical factors in the efficiency of the AD process. The analysis revealed that the MSW sample contained a significant amount of organic material suitable for biogas production, with a balanced moisture content conducive to the anaerobic process. The data obtained from this proximate analysis were crucial in designing the AD process, optimizing system parameters for the specific waste composition, and estimating potential biogas yield and overall efficiency.

### 2.2 Site selection

This study has proposed the location Halishahar for setting up a medium large-scale, decentralized biogas plant. The current dumpsite is located here, and the transports for collecting MSW are kept here. So, additional transport time and distance for MSW transport to the WtE plant will be minimal. The total area of the location is 9.64 km^2^. Around 155 k people live in this area. Considering the 0.41 kg/day per capita waste generation rate and 42 % waste collection efficiency, as shown [23], around 27 TPD MSW (around 20 TPD organic fraction of MSW) can be obtained from this area.

**Fig. 4:**
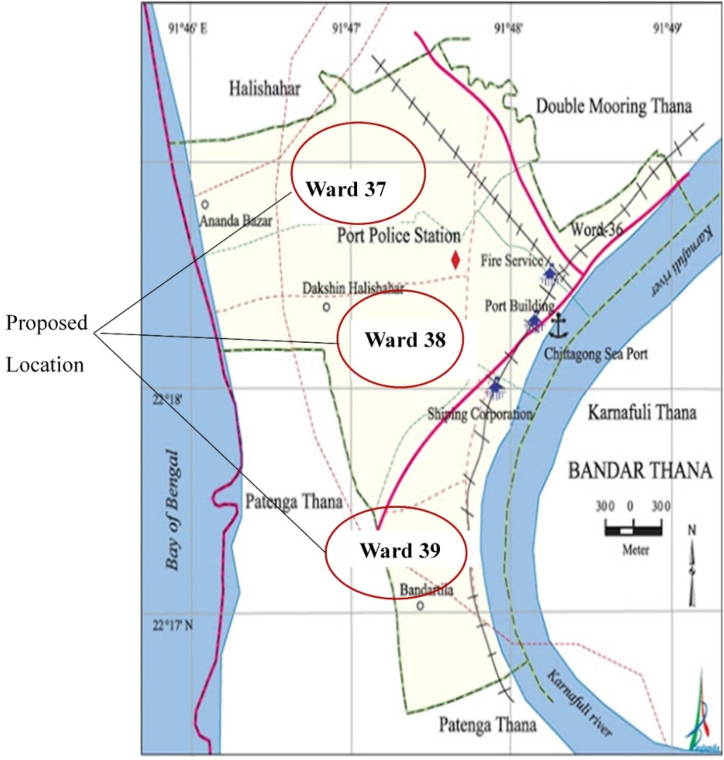
Proposed location for the present study.

There is a compost plant in Halishahar that processes 5 TPD organic fraction of MSW (OFMSW) to produce organic fertilizer. Therefore, around 15 TPD OFMSW are available for processing in the WtE plant. This study has proposed three decentralized biogas plants, each having a handling capacity of 5 TPD OFMSW. Three potential sites for setting up the plants are shown in Fig. 4.

### 2.3 Determination of design parameters for anaerobic digester

According to Nirapod Engineering Limited, continuous, mesophilic wet digestion systems are used for biogas production in Bangladesh due to low solid content, tropical climate conditions and economic prospects. Until 1992, Indian floating dome type biogas plants were used, but all those plants have gone out of order within 3–4 years due to corrosion. In 1992, the Chinese fixed dome type model was introduced, and after that, the use of the Indian floating dome type completely stopped. Most of the digesters in Bangladesh are now fixed dome type digesters, although the largest agro-industrial biogas plant is CSTR type. Up to 600 m^3^ of biogas digester (fixed dome type) can be built using local technology, experts, and materials. Above 600 m^3^, foreign experts are needed to design and construct biogas plants. For this, investment cost/m^3^ increases to a great extent. Considering investment cost, availability of local experts, ease of construction and maintenance, level of use of local materials and resources, level of similar usage and performance records in Bangladesh and adaptability to future situations, this study has considered medium large-scale fixed dome digester for further analysis.

Table 2 illustrates the design parameters which were considered based on the suggestions of local experts.

**Table 2:** Design parameters.

Parameters	Values considered	Units
TS (Total Solid)	8	%
Temperature	30	^o^C
pH	7.2	–
Carbon to Nitrogen Ration (C/N)	25/1	–
HRT	30	Days

assumptions made for volume calculation of the fixed dome digester is shown in Table 3 [26]. Whereas Table 4 shows the assumptions made for calculating the geometrical dimensions of the digester.

**Table 3:** Assumptions for volume calculation [28].

The assumption for volume calculation	Description
Vc = 5 % V	V_c_ = Volume of gas collecting chamber
Vs = 15 % V	V_S_ = Volume of sludge layer
Vgs + V_f_ = 80 % V	V_gs_ = Volume of gas storage chamber V_f_ = Volume of fermentation chamber
Vgs = V_H_	V_H_ = Volume of hydraulic chamber
Vgs = 0.5 (Vgs + V_f_ + Vs) K	K = Gas production rate per m^3^ digester volume per day. For Bangladesh, K = 0.4

**Table 4:** Assumptions for geometrical calculation [28].

The assumption for geometrical calculation	Description
D = 1.3078 X V^1/3^	D = Internal diameter of the digester
V_1_ = 0.0827 D^3^	V_1_ = Volume of the upper dome
V_2_ = 0.05011 D^3^	V_2_ = Volume of the bottom
V_3_ = 0.3142 D^3^	V_3_ = Volume of the fermentation chamber
R_1_ = 0.725 D	R_1_ = Curvature radius of the dome
R_2_ = 1.0625 D	R_2_ = Curvature radius of the bottom
f_1_ = D/5	f_1_ = Vector rise of the dome
f_2_ = D/8	f_2_ = Vector rise of the bottom

### 2.4 Techno-economic analysis

Many researchers have used Aspen Plus® method to evaluate the feasibility of waste-to-energy projects [[27], [28], [29]]. Aspen Plus® is one of the most powerful frameworks for optimization, architecture, sensitivity, and economic assessments compared to other process simulators. However, Aspen Plus® has a very high license cost ($2000/year for university purposes) and needs a certain degree of interface experience [[30], [31], [32]]. In contrast with Aspen Plus®, RETScreen Software, in many cases, is preferred by many researchers [33,34]. The key benefit of this software is that the user can customize the calculation sheets, making user-tool interaction easier. Furthermore, this free decision-making tool is designed to assess the feasibility and performance of renewable energy and energy efficiency programs. Considering the benefits, this research used RETScreen software to conduct the techno-financial analysis of the proposed power plant. RETScreen is a decision-making and capacity-building tool that several researchers have successfully used to assess various renewable energy projects. The program can conduct a five-step standard analysis, which includes an energy model, cost analysis, GHG analysis, financial analysis, and sensitivity and risk analysis. Based on the local site condition and device characteristics, the energy model estimates annual energy output by the proposed design. Pre-tax and after-tax cash flows, interest payments, income tax, asset depreciation, and financial viability metrics are all concepts used in financial analysis. The effect graph, Monte Carlo simulation, median and confidence interval, and risk analysis model validation are all part of the sensitivity and risk analysis. It is possible to assess the feasibility of a proposed design by conducting both of these analysis.

### 2.5 Quality control and quality assurance

Rigorous quality control (QC) and quality assurance (QA) measures were integral to the research methodology, ensuring the reliability and validity of the collected data. This encompassed a comprehensive evaluation of waste sampling and analysis techniques to reflect MSW's composition and volume accurately. Additionally, the site selection for the AD facility was critically assessed, along with the design and financial aspects of the project, ensuring adherence to established standards and practices. Applying QC and QA protocols underpinned the credibility of the study's recommendations for implementing AD technology in Chittagong, aligning with sustainable waste management and energy production goals. This detailed analysis underscores the feasibility and strategic importance of deploying AD technology in Chittagong, backed by a systematic approach to data collection, site selection, and adherence to quality standards. The proposed biogas plants in Halishahar exemplify a sustainable solution to MSW management challenges, contributing to environmental sustainability and achieving global sustainability targets in Bangladesh.

### 2.6 Environmental analysis

This study used the IPCC Guidelines for GHG inventory levels to estimate emissions GHG calculation tool was used to perform the calculation [28]. The GHG emissions calculation tool is a free Excel-based tool developed by the Greenhouse Gas Protocol and the World Resources Institute to help people measure their GHG emissions using the GHG Protocol.

## 3 Results and discussion

### 3.1 Design of proposed anaerobic digestion system

**Fig. 5:**
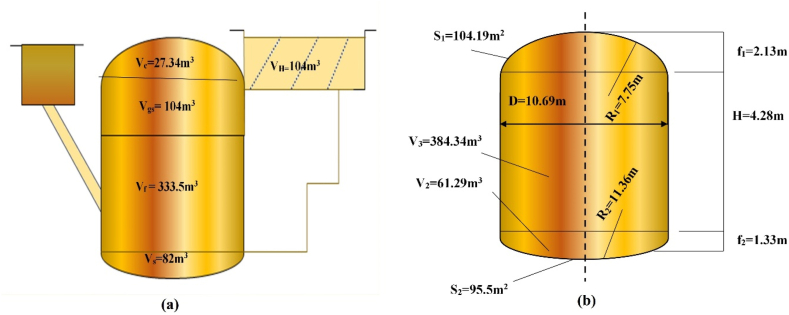
(a) Cross section of the proposed digester; (b) Geometric dimensions of the proposed digester.

Table 5 summarizes the anaerobic digestion system design parameters considered in this study. The study suggested that 5 TPD segregated OFMSW would be diluted with 7.5 m^3^ water to get an 8 % concentration of TS, which is considered a favorable condition for digestion. Fig. 5a shows the cross section and Fig. 5b presents the geometric dimensions of the proposed digester. For a tropical environment with an average air temperature of 35 °C, an HRT of 30–40 days is suggested. A retention period of 35 days is used in this analysis, suggesting that this plant would need a 437.5 m^3^ active reactor volume. The study has assumed that 20 % of 5 Ton is dry matter. The Volatile Solids (VS) content of the dry matter is 80 %, meaning that VS amounts to 800 kg of the 12.5 m^3^ of diluted feedstock, and the share of VS amounts to 64 kgVS/m^3^.

**Table 5:** Anaerobic digestion system design parameters.

Parameters	Values
Operating Temperature	35 °C
Operating Stage	Mesophilic stage
Digester Volume	547 m^3^
Feedstock	Food waste and water
Feed/day	5 TPD
PH	7.2–7.5
Organic loading rate (OLR)	1.82
Total solid content	20 %
Volatile solid	80 %
C/N ratio	25/1
Solid Retention time	35 days
Methane content	60 %
Biogas potential	536 m^3^

As a result, the organic loading rate of the system would be 1.82 kgVS/m^3,^ which is acceptable for a non-stirred AD system (the organic loading rate should be below 2 kg VS/m^3^ for a non-stirred AD system) [28]. The design and obtained parameters of the fixed dome digester are shown in Fig. 4 based on the calculations in the Appendix. Assuming that OFMSW normally produces 0.67 m^3^ of biogas per kgVS (considering 60 % methane content and 0.4 m^3^ CH_4_/kgVS), approximately 536 m^3^ of biogas should be produced per day (1.82 kgVS/m^3^day × 0.67 m^3^/kgVS × 437.5 m^3^). Since 60 % of the biogas is methane (CH_4_), the methane yield is 321.6 m^3^/day. According to Rahman Renewable Energy Co. Ltd., Bangladesh, to produce 1 kWh of electricity, a typical 100 % biogas generator requires 0.707 m^3^ biogas. So, to run a 50 kW gas generator for 12 h, 424 m^3^ biogas would be needed. The rest of the biogas (112 m^3^ biogas) can provide biogas to 44 households for cooking purposes.

### 3.2 Financial evaluation of the proposed design

A summary of the financial evaluation of the proposed design is shown in Table 6. The financial analysis of an anaerobic digestion plant project reveals its profitability and quick returns on investment. The project's initial costs are $98,640, with no fuel costs associated with the operation. The savings are $19,899. The debt payments are $16,840, reflecting a net saving in the first year. The project generates electricity export revenue of $30,240, and no revenue from GHG reductions. The total annual savings and revenue are $30,240, and the net annual cash flow is $33,299.

**Table 6:** Financial viability.

	Financial parameters			
	Inflation rate		%	2 %
	Project life		Yr	10
	Debt ratio		%	70 %
	Debt interest rate		%	7 %
Debt term			Yr	5
Costs | Savings | Revenue				
**Initial costs**				
	Initial cost	100 %	$	98,640
	**Total initial costs**	**100 %**	**$**	**98,640**

**Yearly cash flows - Year 1**				
	**Annual costs and debt payments**			
	Fuel cost - proposed case		$	0
	O&M costs (savings)		$	−19,899
	Debt payments - 5 yrs		$	16,840
				
	**Total annual costs**		**$**	**−3059**
	**Annual savings and revenue**			
	Electricity export revenue		$	30,240
	GHG reduction revenue		$	0
	Other revenue (cost)		$	0
	CE production revenue		$	0
	**Total annual savings and revenue**		**$**	**30,240**
**Net yearly cash flow - Year 1**			**$**	**33,299**
**Financial viability**				
	Pre-tax IRR - assets		%	38.4 %
	Simple payback		Yr	2
	Equity payback		Yr	0.86

The project's financial viability is measured by a 38.4 % pre-tax IRR - Assets, indicating its high profitability. The simple payback period of two years and the equity payback period of 0.86 years are noteworthy, as only a part of the investment is from equity due to the 70 % debt ratio. However, this simplified analysis does not consider potential market conditions, operational risks, or other financial factors such as taxes or changes in government incentives.

In conclusion, the project is highly profitable with quick returns on investment due to the lack of fuel costs and the ability to generate significant revenues from electricity export.

### 3.3 Environmental impact of proposed design

This study performed a life cycle assessment of the 5 TPD biogas plant using the GHG emission calculation tool. A summary of the analysis is shown in Table 7. The table presents a summary of greenhouse gas (GHG) emissions associated with the operational activities of an anaerobic digestion (AD) plant, as well as the emissions avoided by this process compared to conventional waste management and energy production methods. The table includes emissions from operational activities (8.62 kg CO_2_-eq/ton of organic waste), unavoidable leakages (21.0 kg CO_2_-eq/ton of organic waste), direct GHG emissions from AD (29.62 kg CO_2_-eq/ton of organic waste), avoided GHG emissions from electricity production (406.07 kg CO_2_-eq/ton of organic waste), and avoided GHG emissions from organic waste landfilling (630.00 kg CO_2_-eq/ton of organic waste).The net GHG emissions from AD (−1006.45 kg CO_2_-eq/ton of organic waste) represent the net impact of the AD process, taking into account both direct emissions and emissions avoided by generating energy and preventing landfill gas emissions. The total GHG reduction from AD per month (−140.96 tons CO_2_-eq/month) equates to the entire volume of waste treated by the facility in one month. Recycling can also prevent GHG emissions compared to producing goods from virgin materials, reducing emissions per ton of recyclables processed. The total monthly GHG emissions saved through recycling activities at the facility is −78.21 tons CO_2_-eq/month.

**Table 7:** GHG emissions.

Activity	GHG Emissions	Unit
GHG emissions from operational activities	8.62	kg of CO_2_-eq/ton of organic waste
GHG emissions through unavoidable leakages	21.0	kg of CO_2_-eq/ton of organic waste
Direct GHG emissions from anaerobic digestion	29.62	kg of CO_2_-eq/ton of organic waste
Avoided GHG emissions from electricity production	406.07	kg of CO_2_-eq/ton of organic waste
Avoided GHG emissions from organic waste landfilling	630.00	kg of CO_2_-eq/ton of organic waste
Net GHG emissions from anaerobic digestion (life cycle perspective)	−1006.45	kg of CO_2_-eq/ton of organic waste
Total GHG reduction from anaerobic digestion per month	−140.96	ton of CO_2_-eq/month kg of CO_2_-eq/ton of mixed recyclables ton of CO_2_-eq/month
Net GHG emission from recycling (life cycle perspective)	−790.01	
Total GHG reduction from recycling per month	−78.21	

### 3.4 Overview of proposed WtE recovery option for chittagong

**Fig. 6:**
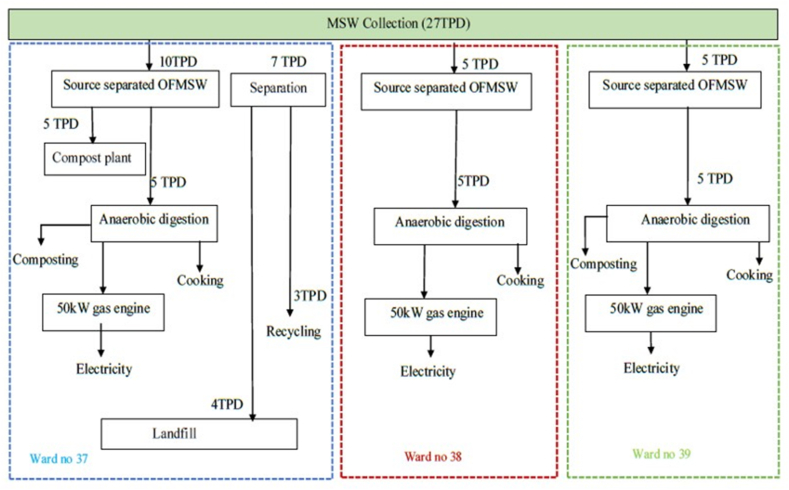
Proposed WtE recovery option for Chittagong.

Fig. 6 shows the proposed WtE recovery option for Chittagong. Around 27 TPD MSW is being generated in the proposed location, Halishahar. Among these, around 20 TPD are organic and 3.3 TPD are recyclable. At present, there is a compost plant that collects 5 TPD source separated organic waste and feeds it to the plant. Therefore, around 15 TPD organic waste is still present in the main waste stream. This study proposed building three decentralized biogas plants in Ward 37, 38, and 39.

The dumpsite is situated in Ward 37 and all the dump trucks are kept there. Therefore, transportation distance and time will be comparatively lower if the plants are constructed on these sites. For Ward 37, 10 TPD sources separated organic waste to be collected to feed the compost and biogas plants. In other Wards, 5 TPD source separated waste are to be collected. Chittagong City Corporation can involve NGOs for source separate waste collection and preprocessing. Around 3.3 TPD of the waste steam is recyclable. The informal sector generally handles this recycling in Chittagong. Around 4 TPD waste is inorganic and non-recyclable, which can be dumped in the current dumpsite. Around 24 TPD waste (the informal sector separates recyclable products) is dumped from the Halishahar area without a waste treatment facility. AD can reduce waste volume by 90 % and CO_2_-equivalent emissions by 500 tons per month. This is due to the breakdown of organic waste, producing biogas and digestate, which have lower volumes than the original waste. The 90 % figure is based on empirical data from specific facilities and varies based on waste composition, digester efficiency, and retention time. Anaerobic digestion captures methane, a potent greenhouse gas, preventing its release and allowing it to be used as renewable energy. The 500 tons of CO_2_-eq/month saved is calculated based on the amount of methane emitted by landfilling the waste minus CO_2_ emissions from combusting the methane collected via digestion.

### 3.5 Comparative analysis

The comparative analysis and critical review across four studies are shown in Table 8.

**Table 8:** Comparative analysis and critical review across four studies.

Parameter	This study	Study 1 [35]	Study 2 [36]	Study 3 [37]
Focus	AD process for MSW treatment, local digester design, techno-financial, and environmental analysis	Financial analysis of AD and gasification WTE plants	Biogas upgrading, power requirements of biogas upgrading	Feasibility of co-AD plant at the local scale
Location	Chittagong, Bangladesh	South Africa	Not specified	Urban district (not specified)
MSW Treatment	Source-separated organic MSW	AD and gasification technologies	Biogas upgrading technologies	Co-AD plant parameters
Pre-treatment	None	Not specified	Not specified	Not specified
Financial Analysis	Pre-tax IRR – assets, Simple payback, Equity payback	NPV, IRR, PI, PBP, LCOE, LCOW analysis	Comparative analysis of various plant configurations	Economic analysis with LCOW index
Initial Cost	$98,640	Not specified	Not specified	Not specified
Revenue	Electricity export, savings, and revenue	NPV for highest scenario	Not specified	Not specified

### 3.6 Critical analysis

The comparative analysis across the four studies reveals varied approaches to WtE technologies. Our study is unique in its local context application, specifically tailored for Chittagong, Bangladesh, focusing on source separated organic MSW. Study 1 and Study 3 also focus on financial aspects but with different technological approaches, namely AD, gasification, and co-AD plants. Study 2 diverges by concentrating on biogas upgrading and power requirements, highlighting the diversity in addressing WtE challenges. Each study has its unique focus and context, with our study standing out for its detailed techno-financial and environmental analysis, specifically for a developing country. The financial aspects are a common theme, with Study 1 providing a detailed financial model assessment, including NPV and IRR, similar to our approach. Study 2 and Study 3 contribute to the broader picture by evaluating biogas upgrading and the feasibility of Co-AD plants, respectively. The critical review indicates that while each study offers valuable insights, our work is particularly significant for its practical application in a developing country context, making it a notable contribution to sustainable waste management.

### 3.7 Practical application

The study presents a comprehensive framework for implementing AD technology in managing MSW, focusing on the organic fraction. It emphasizes the importance of source-separated organic waste for optimal AD functioning, providing a roadmap for waste management authorities. The study proposes medium-large fixed dome digesters designed to suit local expertise and materials, facilitating the establishment of decentralized biogas plants that ensure efficient waste-to-energy conversion. The research also highlights the financial feasibility of AD-based energy production, indicating potential revenue generation through biogas utilization. The economic analysis underscores the attractiveness of these plants for investors, with a relatively short payback period. The study also highlights the potential environmental impact mitigation, with a 93 % reduction in GHG emissions compared to coal-fired power plants. The proposed plants could positively impact local communities by providing biogas for household cooking and reducing waste volumes. This model can be replicated for developing nations facing MSW management challenges, offering a comprehensive approach from waste composition analysis to financial feasibility. Policy recommendations are supported, emphasizing the importance of waste segregation practices. Encouraging authorities to prioritize source-separated organic waste for AD plants could drive better waste management policies. The research's outcomes serve as educational material, aiding in awareness campaigns about sustainable waste management practices. In conclusion, the practical applications of this research extend beyond the technical realm, impacting waste management policies, economic sectors, community engagement, and environmental stewardship. Implementing these insights could pave the way for a more sustainable and eco-friendly future.

## 4 Conclusion and Future Directions

This study has undertaken a comprehensive techno-economic and environmental analysis of implementing medium to large-scale decentralized anaerobic digestion (AD) plants for municipal solid waste (MSW) in Chittagong, Bangladesh. Our findings illuminate the path for sustainable waste management and energy production, offering a nuanced understanding of the potential and challenges associated with waste-to-energy (WtE) conversions in developing urban contexts. Our research makes a significant contribution to the scientific community by providing empirical evidence on the viability of AD technology in efficiently managing organic MSW and producing renewable energy in a setting like Chittagong. We have designed a feasible model for AD plants that leverages local waste characteristics and climatic conditions, enhancing the practicality and sustainability of WtE solutions. The study's scientific value lies in its detailed analysis of operational parameters, environmental impacts, and economic viability, presenting a holistic view of AD's potential to address waste management and energy challenges simultaneously. By demonstrating the technical feasibility, environmental benefits, and economic attractiveness of AD plants, our study provides a blueprint for policymakers, urban planners, and renewable energy stakeholders interested in adopting sustainable waste management practices. The model proposed can serve as a replicable framework for similar urban areas in developing countries, where waste management remains a critical issue, and the quest for renewable energy sources is ongoing. The success of the proposed AD plants heavily relies on effective source separation of organic waste, which may require significant behavioral changes and community engagement efforts. Additionally, our analysis is constrained by the assumptions made regarding technological performance and market conditions, which may evolve over time.

Building on the foundation laid by this study, future research should explore innovative approaches to enhance the efficiency and scalability of AD technology. Investigating alternative pre-treatment methods, optimizing digester design for varied waste compositions, and assessing the long-term operational challenges would be valuable. Moreover, further studies could examine the socio-economic impacts of AD plant implementation, including job creation, public health benefits, and community acceptance. Collaborative research involving multi-disciplinary teams could also explore the integration of AD systems within broader waste management and renewable energy frameworks, maximizing the environmental and societal benefits. This study underscores the scientific and practical merits of deploying AD technology for MSW management in Chittagong, offering insights that contribute to the global discourse on sustainable urban development and renewable energy. By highlighting the opportunities, addressing the challenges, and outlining the path for future inquiry, this research advances our collective understanding of waste-to-energy solutions as a pivotal component of sustainable urban ecosystems.

## Institutional review board statement

Not applicable.

## Informed consent statement

Not applicable.

## Data availability statement

Not applicable.

## CRediT authorship contribution statement

**Samina Alam:** Writing – original draft, Visualization, Validation, Software, Methodology, Formal analysis, Data curation, Conceptualization. **Md. Rokonuzzaman:** Writing – review & editing, Visualization, Supervision, Project administration, Funding acquisition. **Kazi Sajedur Rahman:** Writing – review & editing, Supervision, Project administration, Funding acquisition. **Akramul Haque:** Writing – review & editing, Visualization, Validation. **Md Shahariar Chowdhury:** Visualization, Validation. **Tofan Agung Eka Prasetya:** Visualization, Validation, Funding acquisition.

## Declaration of competing interest

The authors declare that they have no known competing financial interests or personal relationships that could have appeared to influence the work reported in this paper.
