# Supplementary material for: Techno-economic and environmental analysis of organic municipal solid waste for energy production
Source: Heliyon. 2024 May 21;10(11):e31670. doi: 10.1016/j.heliyon.2024.e31670 (PMC11145329; doi:10.1016/j.heliyon.2024.e31670)
Supplement: Multimedia component 1 [file mmc1.docx]

APPENDIX

TECHNO-ECONOMIC AND ENVIRONMENTAL ANALYSIS

## C1 Design of Anaerobic Digestion System

Let HRT = 35 days

Total Organic Waste =5000kg

In 8% concentration of TS (To make favorable condition)

8 Kg. Solid = 100 Kg. Influent

1 Kg. Solid = 100 / 8 Kg influent

5000 Kg Solid = 100 x 5000/ 8 = 12500

Total influent required = 12500 Kg

Water to be added to make the discharge 8% concentration of TS =12500 Kg - 5000 Kg. = 7500 Kg.

Working volume of digester = Vgs +V_f_ =Q.HRT= 12500 x 35/1000 =437.5m^3^

From geometrical assumptions-

Vgs + V**_f_** = 0.80 V= 437.5 m^3^

V=547m^3^

D = 1.3078 V^1/3^

Therefore, D=10.7 m

Now,

| f_1_ | = D/5 = 10.7 /5 = 2.14 m |
| --- | --- |
| f_2_ | = D/8 = 1.33m |
| R _1_ | = 0.725 D = 7.75 m |
| R_2_ | = 1.0625 D = 11.36 m |
| V_1_ | = 0.0827 D^3^ = 101.16 m^3^ |
| Vc | = 0.05V = 27.34 m^3^ |

Vs = .15V= 83.02 m^3^

V_gs_ = 0.50 x (Vgs + Vf + Vs) x K =.5x (437.5+83.02) x.04= 103.9 m^3^

Again,

| V_1_= [{(Vc + Vgs) - {p xD^2^x H_1_}/4]  From this, we get H_1_=.334m  If h= 3.8m  We get, h = h_3_ + f_1_ + H_1,_  or, h_3_= 1.32 m  Again, we know that  V_gs_ = V_H_  Or, 103.9 m^3^= 3.14x(D_H_)^2^xh_3_/4  D_H_ = 10m |
| --- |

**C2 Biogas for Cooking**

Gas demand per person and meal: 0.15 m³ biogas

Gas demand per meal: 1.2 m³ biogas

Cooking-energy demand: 2.4 m³ biogas

Consumption rate of gas burner: 175 l/h per flame (2-flame cooker)

Operating time: (2 × 3 h) + 1 h for tea

Biogas demand: 7 h × 350 l = 2.5 m³

**C3 Estimation of Capital Investment Cost**

**Table C1 Estimated budget requirement for total installation of the proposed plant**

| **No** | **Materials** | **Amount** | **Rate**  **BDT** | **Total**  **BDT** |
| --- | --- | --- | --- | --- |
| 1 | Brick 1^st^ Class | 45000 | 9.00 | 405000.00 |
| 2 | Sand | 12000 cft | 15.00 | 180000.00 |
| 3 | Concrete | 3500 cft | 100.00 | 350000.00 |
| 4 | Rod (8 mm, 10 mm & 12mm) | 8500 kg | 60.00 | 510000.00 |
| 5 | Cement | 750 bags | 425.00 | 318750.00 |
| 6 | Poly paper | 25 kg | 120.00 | 3000.00 |
| 7 | Plastic Paint | 80 kg | 200.00 | 16000.00 |
| 8 | Best quality 8’’dia PVC pipe | 40 ft | 300.00 | 12000.00 |
| 9 | GI Wire (No 24 &10) | 200 kg | 120.00 | 24000.00 |
| 10 | Equipment**:** Mixer device  Center pipe  Delivery flexible pipe  Gas Valve RB- Italy  Nipple  Biogas stove-40 prices  GI nipple both pass  Tap  GI Clam |  | 100000.00 | 100000.00 |
| 11 | Earth digging & filling | | | 400000.00 |
| 12 | Biogas Trained Mason | | | 1500000.00 |
| 13 | 50 KW reciprocating engine-generator | | | 2000000.00 |
| 14 | Consultancy Fee- Rahman Renewable Energy Co. Ltd. | | | 750000.00 |
|  | **Total Cost** | | | **6568750.00 BDT** |
|  |  |  |  | **98682.73 $** |

Source: Rahman Renewable Energy Co. Ltd, personal communication, March 25,2021

**C2 Estimation of Operating Cost**

**Table C2 Estimated Salary**

| Staff | No | Salary per month (BDT) | Net salary  (BDT) | Net salary/year |
| --- | --- | --- | --- | --- |
| Operators | 4 | 10000 | 40000 | 480000 |
| Security guard | 2 | 10000 | 20000 | 240000 |
| Driver | 1 | 10000 | 10000 | 120000 |
| Project manager | 1 | 22000 | 22000 | 264000 |
| Total annual cost | | | | 2153022.5 BDT |
|  |  |  |  | 32344.99$ |

**Oil Cost:**

| Diesel oil cost = | 65 (BDT/liter) |
| --- | --- |
| Per trip oil required= | 8 (liter) |
| No of trips= | 2 |
| No of days= | 28 |
| Oil cost per month = | 29120 (BDT) |
| Oil cost per year = | 349440 (BDT)  =5249.66 $ |

**Water Cost:**

| Water cost = | 30.3 BDT/m^3^ |
| --- | --- |
| Water requirement = | 7.5 (m3/day) |
| Water requirement= | 210 (m3/month) |
| Annual Water bill cost = | 2520 BDT  =37.86$ |

Packaging cost /year= 500000 BDT = 7511.53 $

Miscellaneous cost: = Assumed that 10% × 30% of construction cost of Digester

= 197062.5 BDT =2960.48 $

Total Operating Cost = Salary of Employee + Oil cost + Water cost + Packaging cost + Miscellaneous cost= 2153022.5 BDT = 32344.99 $

**Profit from Bio-slurry Sale**

Annual Slurry Generation Rate= 2144kg

Cost of per kg of bio-slurry =5.5 BDT

Profit from Fertilizer sale = 3962112 BDT= 59523.05 $

**Profit from Biogas Sale**

Annual additional biogas generation = 37632 m^3^

Cost of biogas =15BDT

Profit from biogas sale = 564480 BDT =8480.22 $

Saving = Profit from biogas sale+ Profit from bio-slurry – O&M cost = 19898.75 $

**Profit from Electricity Sale**

**Table C3 Revenue from Electricity Sale**

|  | Electricity exported to grid kWh | Revenue  $ |
| --- | --- | --- |
| Proposed Case | 201,600 | 30,240 |

**Figure C1 Cumulative Cash flow**

**
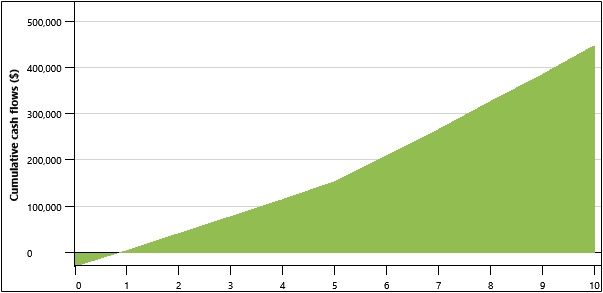
**

**Figure C2 Annual Cash flow**

**
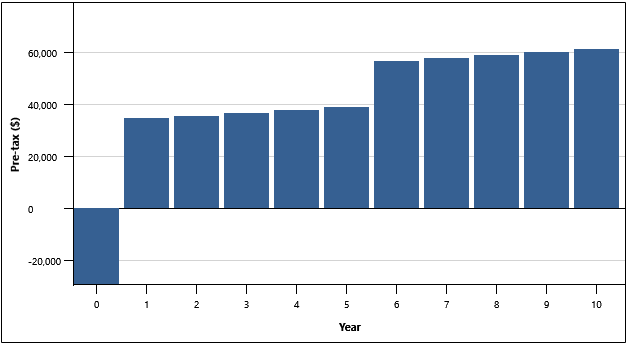
**

**Table C4 Yearly Cash flow**

| **Year** | **Pre-tax** | **Cumulative** |
| --- | --- | --- |
|  | **$** | **$** |
|  |  |  |
| 0 | -29,592 | -29,592 |
| 1 | 34,301 | 4,709 |
| 2 | 35,324 | 40,033 |
| 3 | 36,367 | 76,401 |
| 4 | 37,432 | 113,832 |
| 5 | 38,517 | 152,349 |
| 6 | 56,464 | 208,814 |
| 7 | 57,594 | 266,407 |
| 8 | 58,746 | 325,153 |
| 9 | 59,920 | 385,073 |
| 10 | 61,119 | 446,192 |

**Figure C3 GHG Emission Calculation for landfilling**

**
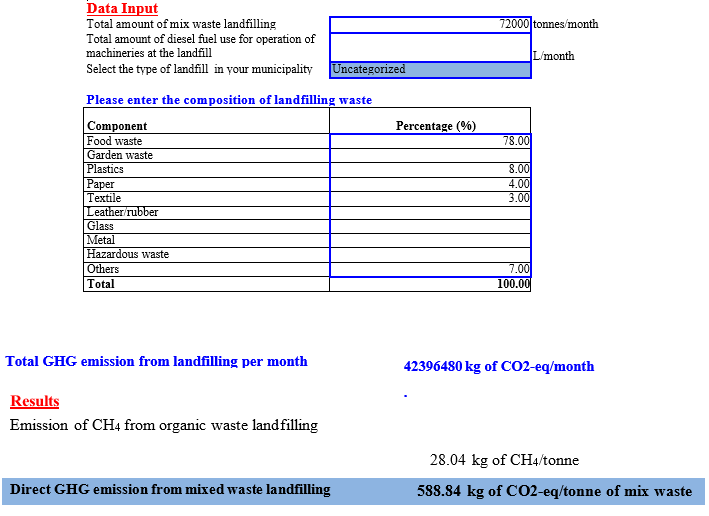
**

**Figure C4 GHG Emission Calculation for Transportation**

**
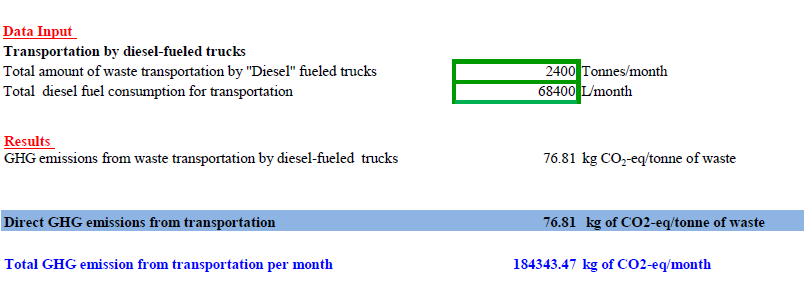
**
